# Amikacin-Loaded Chitosan Hydrogel Film Cross-Linked with Folic Acid for Wound Healing Application

**DOI:** 10.3390/gels9070551

**Published:** 2023-07-06

**Authors:** Yasir Mehmood, Hira Shahid, Numera Arshad, Akhtar Rasul, Talha Jamshaid, Muhammad Jamshaid, Usama Jamshaid, Mohammad N. Uddin, Mohsin Kazi

**Affiliations:** 1 Department of Pharmaceutics, Faculty of Pharmaceutical Sciences, Government College University Faisalabad, Faisalabad P.O. Box 38000, Pakistan; akhtar.rasul@gcuf.edu.pk; 2 Riphah Institute of Pharmaceutical Sciences (RIPS), Riphah International University Faisalabad, Faisalabad P.O. Box 38000, Pakistan; 3 Department of Pharmacology, Faculty of Pharmaceutical Sciences, GC University Faisalabad, Faisalabad P.O. Box 38000, Pakistan; hira-shahid@yahoo.com; 4 Department of Pharmacy, COMSAT University Islamabad, Lahore Campus, Lahore P.O. Box 54000, Pakistan; drnumeraarshad@cuilahore.edu.pk; 5 Department of Pharmaceutics, Faculty of Pharmacy, The Islamia University of Bahawalpur, Bahawalpur 63100, Pakistan; talhajamshaid007@gmail.com; 6 Faculty of Pharmaceutical Sciences, University of Central Punjab, Lahore P.O. Box 54000, Pakistan; pharmchair@hotmail.com (M.J.); usama.jamshaid042@gmail.com (U.J.); 7 College of Pharmacy, Mercer University, 3001 Mercer University Drive, Atlanta, GA 30341, USA; uddin_mn@mercer.edu; 8 Department of Pharmaceutics, College of Pharmacy, King Saud University, P.O. Box 2457, Riyadh 11451, Saudi Arabia

**Keywords:** networks, pH responsive, chitosan polymer, wound dressing, hydrogel film

## Abstract

Purpose: Numerous carbohydrate polymers are frequently used in wound-dressing films because they are highly effective materials for promoting successful wound healing. In this study, we prepared amikacin (AM)-containing hydrogel films through the cross-linking of chitosan (CS) with folic acid along with methacrylic acid (MA), ammonium peroxodisulfate (APS), and methylenebisacrylamide (MBA). In the current studies, an effort has been made to look at the possibilities of these materials in developing new hydrogel film wound dressings meant for a slow release of the antibiotic AM and to enhance the potential for wound healing. Methods: Free-radical polymerization was used to generate the hydrogel film, and different concentrations of the CS polymer were used. Measurements were taken of the film thickness, weight fluctuation, folding resistance, moisture content, and moisture uptake. HPLC, FTIR, SEM, DSC, and AFM analyses were some of the different techniques used to confirm that the films were successfully developed. Results: The AM release profile demonstrated regulated release over a period of 24 h in simulated wound media at pH 5.5 and 7.4, with a low initial burst release. The antibacterial activity against gram-negative bacterial strains exhibited substantial effectiveness, with inhibitory zones measuring approximately 20.5 ± 0.1 mm. Additionally, in vitro cytocompatibility assessments demonstrated remarkable cell viability, surpassing 80%, specifically when evaluated against human skin fibroblast (HFF-1) cells. Conclusions: The exciting findings of this study indicate the promising potential for further development and testing of these hydrogel films, offering effective and controlled antibiotic release to enhance the process of wound healing.

## 1. Introduction

The largest organ in the body, the skin, serves multiple purposes. Every year, the expense of treating its wounds, such as all types of pressure sores, ulcers, burn wounds, and other abrasive and traumatic wounds, is very significant. As a result, many studies on wound dressings have been performed to develop an effective strategy for wound healing [1,2]. One of the major obstacles to wound healing is the infection caused by bacteria and other microorganisms in the wounded area of the skin [3]. Further problems, such as delayed skin recovery, may occur as a result of this infection.

Researchers have been forced to develop a wide variety of novel drug delivery methods for topical administration as a consequence of the utilisation of cutting-edge technology for the distribution of various therapeutic agents, which has resulted in this requirement. The ratio of active ingredients has been kept significantly within the therapeutic range as a result of the practice of delivering active agents at defined rates and to specific areas in accordance with physiological requirements [4]. Potential drug delivery technologies include liposomes, nanoparticles, dendrimers, niosomes, microspheres, microneedles, micelles, and hydrogels [5]. Due to their hydrophilic polymeric system properties, hydrogel films have seen the most success among the aforementioned drug delivery technologies. Due to the presence of several functional groups, including amino, carboxylic, and hydroxyl groups, on the polymeric network of hydrogels, these hydrogels show certain characteristics, including the ability to absorb water. The therapeutic potential of active drugs can be significantly influenced by the delivery mechanism. Because of reported potential changes in their polymeric network systems that could result in an adequate, controlled, and targeted release of the loaded active moiety, polymeric network carriers have drawn much attention in recent years. In addition, the polymeric networks of these systems continue to make it possible for the active moiety to be accommodated while remaining within the therapeutic range [6]. Among the numerous kinds of polymeric carriers, responsive hydrogel films have been demonstrated to be one of the essential drug delivery techniques. Carbohydrate polymers and related hydrophilic polymers are particularly effective platforms for wound-dressing films among polymeric biomaterials. These polymers have been useful in delivering a variety of therapeutic compounds for therapeutic reasons because they are sensitive to chemical, physical, and biological stimuli [7].

A carbohydrate polymer called chitosan has undergone substantial research in a variety of sectors due to its unique properties, including biodegradability, biocompatibility, availability, etc. [8,9]. Additionally, due to its biodegradability, biocompatibility, anti-infection, antimicrobial, and hemostatic ability features, it can be used widely in biobased and biomedical applications [10,11,12]. However, the majority of these uses for chitosan pertain to its hydrogel forms. The creation of hydrogels by cross-linking techniques, both chemical and physical, is essential [13,14,15]. In a recent publication, researchers discussed the possibility of using hydrogel films based on chitosan, which is a commonly obtained carbohydrate, as a wound-dressing application. Chitosan is used to make hydrogel films. For the purpose of wound healing, they have utilised gentamicin as an antibacterial agent loaded in hydrogel film [12]. In the recent publications, researchers used the same monomers to produce hydrogels, and this monomer has an impact on swelling and polymer solubility [16]. In contrast to the latter type, where networks are created through intermolecular interactions, the chemical type of network is built through covalent bonding. Because of their renewability, nontoxicity, biocompatibility, biodegradability, and supramolecular structure, CS hydrogels are regarded as a suitable matrix platform from a material standpoint [17]. The incorporation of different nanoparticles makes CS-based nanocomposites good candidates in biomedical fields that have attracted considerable attention [18]. Cross-linking and nanoparticle cytotoxicity have recently become major challenges, especially in biomedical applications. Finding cross-linkers that are practical, secure, and have the right cytocompatibility and biodegradability is therefore crucial. Several synthetic cross-links are used in hydrogel preparation, but we used folic acid as a natural cross-linker in our hydrogel film preparation. In previous research, folic acid was used as a conjugate agent for polymers and monomers [12]. Folic acid, which is both an essential part of a healthy diet and one of the B vitamins, has been shown to significantly lower the chance of developing a variety of disorders [19].

Burn wounds, acute surgical wounds, traumatic wounds such as those that arise as a result of an accident, and chronic wounds such as diabetic foot, leg, and pressure ulcers are just a few examples of the various types of wounds that patients may present within a range of different settings. Patients may also have acute wounds after surgery, traumatic wounds such as those that arise as a result of an accident, and burn wounds. All wounds contain microorganisms that are a part of the saprophytic microflora of the skin, which might help the wound heal [20]. Skin is the most exposed part of the body to the environment, and it is easily contaminated with bacteria in wounds. *S. aureus*, beta-hemolytic streptococci, and coryneform bacteria are the three skin pathogens that are found most frequently in open environments. In open wounds, it is very difficult to avoid forming such bacteria. Most people use antibiotic cream and ointments for local use.

In most cases, standard systemic antibiotics are sufficient for treating simple postoperative infections. These antibiotics are safe and effective in simple infections due to their strong bacterial sensitivity, good penetration into well-vascularized tissues, and low host toxicity. However, failures of systemic antibiotic therapy occasionally occur and are linked to high costs and morbidity [21]. Amikacin (AM), a potent commercially available antibiotic medication, is frequently given because it is effective in eliminating a wide range of gram-positive and gram-negative bacteria [22]. The therapeutic local drug concentrations of a slow-release antibiotic formulation administered directly to the infection sites may be maintained, preventing systemic exposure to potentially harmful substances. Selection of resistant organisms at distant locales might possibly be prevented as well [23].

In the current investigation, novel antibacterial flexible polymeric hydrogel films were created by cross-linking polymeric chitosan (CS) with folic-acid-functionalized chitosan. These films were found to be both pliable and resistant to bacterial growth. Folic acid can easily conjugate with polymers and can diffuse into cells, which helps to penetrate AM into infected cells. To date, AM topical dosage forms have been available throughout the world. Therefore, we prepared the first unique dosage form of AM along with a biodegradable polymer to treat wounds. This dosage form also helps to mitigate bacterial resistance in low- to middle-income countries.

We have prepared a hydrogel film formulation with a gradual release that contains the medication AM enclosed within a polymeric drug delivery system. CS that degrades naturally was used in this drug delivery technique. As a slow-release medication administration device, the hydrogel film formulation has been studied by using different analysis techniques.

Additionally, their cytotoxicity toward human skin fibroblast HFF-1 cell lines, in vitro AM release, hemolysis, and antibacterial activity against various bacterial species were investigated. This approach can provide a fresh perspective on how to build a straightforward base for an antibacterial wound dressing.

## 2. Results and Discussion

### 2.1. Determination of Drug Entrapment Efficiency

The AM-loaded hydrogel film (2 × 2 cm²) was weighed and then dissolved in a mixture of ethanol and pH 7.4 phosphate buffer (30:70) to determine the amount of AM [24]. The same technique was used to check each hydrogel film AM-1 to AM-5. The results regarding drug entrapment are listed in Table 1. We found that the AM-5 formulation, in which we added the maximum amount of CS, had the highest drug entrapment. This formulation has been chosen for additional characterizations.

### 2.2. Physicochemical Characterization

Various physicochemical parametric tests were used to evaluate the produced hydrogel films. Weight variation, thickness, and folding endurance are shown in Table 2. The effectiveness and repeatability of the formulation preparation procedure are gauged by evaluation tests. It was determined that the film thicknesses varied between 0.042 ± 0.02 and 0.074 ± 0.04 mm. According to reports, the weight fluctuation varied between 0.432 ± 0.08 and 0.522 ± 0.07 g. The folding endurance was discovered to rise from 340 ± 11 to 490 ± 11 as the chitosan concentration was raised from hydrogel film batch F1 to F5. Additionally, the moisture content and uptake increased from 13.10 ± 1.12 to 16.80 ± 1.27 and 13.10 ± 1.31 to 16.70 ± 1.23, respectively.

### 2.3. SEM and Investigation of Surface Roughness by AFM

**Figure 1:**
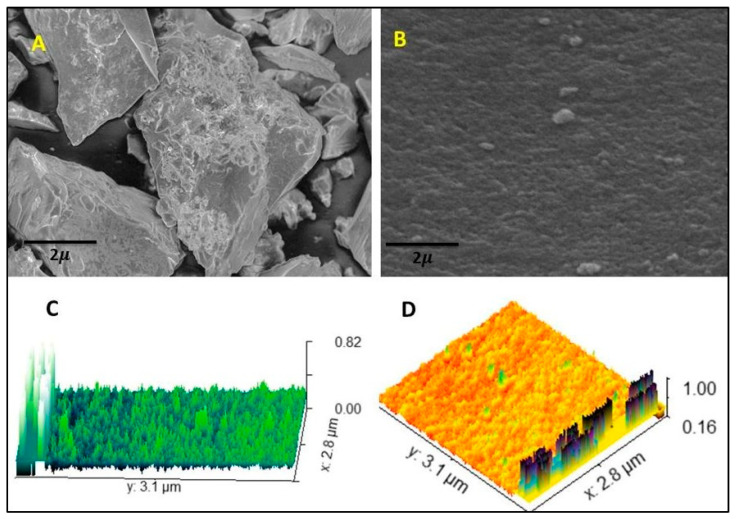
SEM images (**A**,**B**) and AFM images (**C**,**D**) of the hydrogel film.

SEM analysis was used to determine the morphological characteristics of the raw AM and the hydrogel film (AM-5), while the AFM image provides a three-dimensional view of the surface topography, indicating a homogeneous surface with some nanoscale roughness. AM raw and hydrogel film micrographs are shown in Figure 1A,B. The SEM image clearly shows that the hydrogel film made from CS has an uneven surface (Figure 1B). Additionally, roughness can be seen all over the uneven surface, which may be the result of drying treatment and shrinking of the polymeric system. The hydrogel film AMS-5 composition surface roughness was also confirmed by AFM (Figure 1C,D). This roughness may increase when the CS concentration increases. It is possible to modify the structure of the hydrogel film for different applications by changing the amount of polymer and monomers, which can affect the physicochemical properties of the hydrogel film cavities and their interaction with guest molecules.

### 2.4. Fourier Transform Infrared Spectroscopy

**Figure 2:**
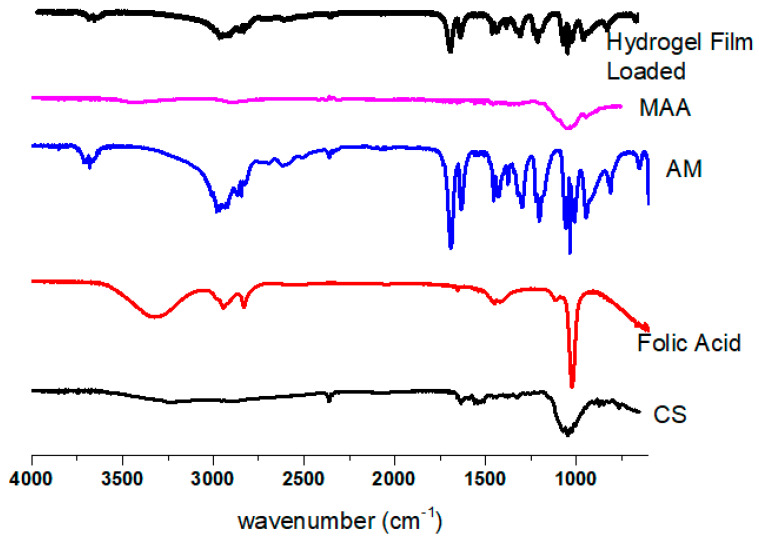
FTIR spectra of AM, CS, MAA, folic acid, and hydrogel film.

AM, CS, MAA, folic acid, and hydrogel film FTIR spectra are shown in Figure 2. Film samples were evaluated using the 600–4000 cm^−1^ scanning range. The AM standard exhibits a characteristic absorption band in the 800–1800 cm^−1^ range. The peaks in the area of 1100 cm^−1^ are caused by the presence of SO_4_ asymmetric stretching vibrations, a counterion to the sulfate ion. The C–O stretching bands of carbohydrates are represented by the absorption band, which is found between 1200 and 1650.23 cm^−1^. N–H single bond stretching vibrations are responsible for the peak at 650 cm^−1^ [25]. The OH, C–H, and C–O stretching frequencies were detected in the FTIR spectra of the CS band at 3456 cm^−1^, 2932 cm^−1^, and 1065 cm^−1^, respectively. On the other hand, bending vibration of -OH groups on the MAA is demonstrated at 1181 cm^−1^ [26]. The FTIR spectrum of folic acid had peaks at approximately 1135 cm^−1^ for carbonyl groups and 1527 cm^−1^ for vinyl groups. A wide band ranging from 3200 to 3400 cm-^1^ was observed for the OH of COOH [27]. The hydrogel film showed peaks at 1678.56 cm^−1,^ which indicated the presence of an AM absorption band. Little displacement was found in this group from 1650.23 to 1678.56 cm^−1^. No folic acid or MAA peaks can be seen in the hydrogel film due to the overlapping of tiny peaks. The characteristic absorption band of CS was also present in the hydrogel film at 2356.32 cm^−1^. The modest displacement in the peaks of the separate components may have been caused by the cross-linking of polymeric chains. These cross-linking peaks proved that a polymeric network had formed.

### 2.5. Thermal Analysis

**Figure 3:**
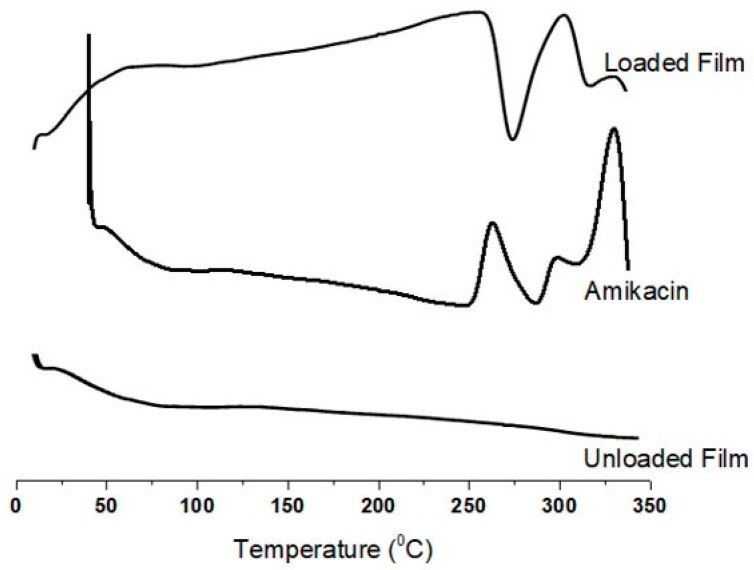
Differential scanning calorimetry (DSC) thermograms of amikacin (AM), unloaded hydrogel film, and loaded hydrogel film.

Figure 3 shows the unloaded hydrogel film, loaded hydrogel film, and AM with heat flow between 0 and 350 °C. In a pure AM DSC graph, two sharp exothermic peaks at approximately 260 °C and 320 °C correspond to the melting point and degradation, respectively. The glass transition temperature is indicated by the endothermic peak at 290 °C. The DSC of the unloaded hydrogel film did not exhibit any distinct peaks. From 100 °C to 350 °C, there was only a very tiny curve from 25 °C to 75 °C, which was caused by polymeric networking breaking. The minor DSC curve that the loaded hydrogel film showed at 50 °C was also caused by the breakdown of the polymeric networking, which was already observed in the unloaded hydrogel film. An exothermic peak was also noted at approximately 260 °C, indicating a rupture in the polymeric network link [28]. The thermogram from the DSC of the hydrogel film formulations (loaded) shows a sharp endothermic peak at 300 °C that represents AM degradation within the polymeric network, although it was observed to be more stable than AM alone. The thermogram clearly shows that AM has more thermal stability in a manufactured hydrogel film than it does in a pure form. The AM polymeric nanoparticles may have better heat stability, according to a previous study [29]. The results demonstrate that AM in the formulated hydrogel film possesses more thermal stability than that of the pure drug.

### 2.6. Powder X-ray Diffraction Analysis

**Figure 4:**
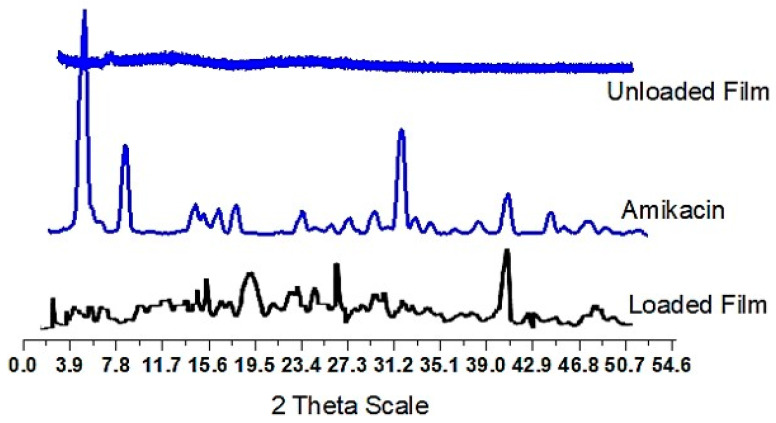
*Powder X-ray diffraction* (PXRD) of AM and unloaded and loaded hydrogel films.

To confirm the amorphous or crystalline nature of the samples shown in Figure 4, XRD of AM hydrogel film (unloaded and loaded) formulations was carried out. Sharp drug peaks at 2θ 3.92°, 7.81°, 15.60°, 26.24°, 32.16°, 40.35°, and 44.21° were observed in the AM sample, which indicated its crystalline nature. When the hydrogel film was loaded, the diffraction pattern revealed the presence of characteristic AM peaks (2θ 15.61°, 19.56°, 40.24°, and 48.41°), which are close to drug-indicated peaks but have been somewhat altered in some regions. This suggests that AM in the loaded hydrogel film is still in its crystalline state. The slight change in peaks also indicated that the medicine (AM) was loaded onto a manufactured hydrogel film in its stable form. Such symptoms suggest that a hydrogel film that meets the criteria for an improved drug delivery system should be employed to disperse drugs. Overall, the DSC thermogram of AM shows a sharp endothermic peak, indicating its melting point. The unloaded hydrogel film and loaded hydrogel film show broad endothermic peaks, indicating the presence of water in the hydrogel matrix. The loaded hydrogel film also shows a small endothermic peak attributed to the presence of AM within the matrix.

### 2.7. Determination of Gel%, Yield% and Gel Time

**Figure 5:**
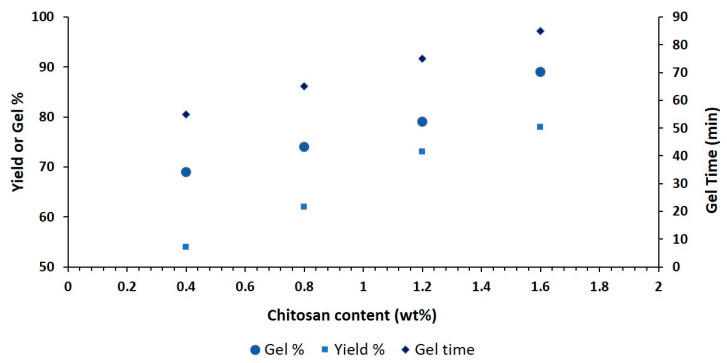
Effect of chitosan hydrogel film on gel%, yield% and gel time.

Figure 5 shows how different ingredients affect the gel percentage, yield percentage, and gel time of the hydrogel film. CS concentration increases lead to higher gel and yield percentages because the polymeric network has the ability to entrap water in it. Gel formation may be due to the availability of additional radicals for polymerization due to an increase in polymer concentration [30]. The formulation’s use of a precise amount of folic acid to cross-link CS with MAA (monomer) may be the cause of the longer gelling time. A greater reaction (polymerization) rate could be the cause of the shorter gelling time [31]. These results are similar to those of previously reported studies [32].

### 2.8. Swelling Study

**Figure 6:**
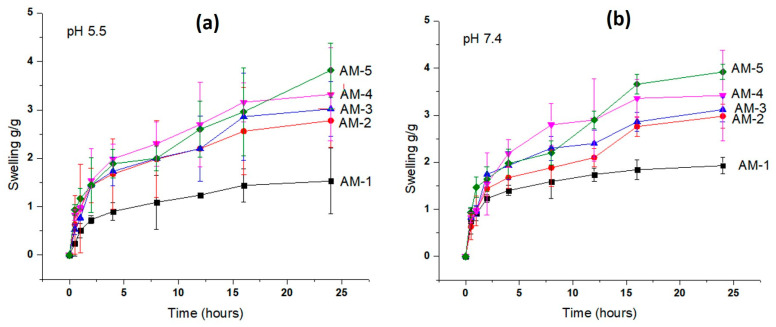
Swelling index of the hydrogel film (AM-1 to AM-5) at pH 5.5 (**a**) and at pH 7.4 (**b**).

The purpose of this test was to evaluate the impact of polymeric network components on the performance of hydrogel film swelling. pH levels of 5.5 and 7.4 were selected because of the typical sick skin states. The swelling of the hydrogel film was recorded at pH 5.5 and 7.4, as shown in Figure 6a. Compared to that at pH 5.5, swelling at pH 7.4 was increased due to more functional group (carboxyl) ionization. Complete ionization starts ion repulsion, which causes swelling to grow [33]. The maximum swelling index was observed at pH 5.5 (3.78 g within 24 h), as shown in Figure 6a. At pH 7.4, a swelling index of 3.92 g was observed, which was near pH 5.5 (Figure 6b). The highest swelling index played a role in the high drug release. From the graph, it was seen that the concentration of CS increased the swelling of the hydrogel film. This increased swelling is presumably because more radicals are available due to an increase in polymer concentration [34]. Additionally, CS alters the degree of cross-linking and carboxymethylation, which results in hydrophilicity by weakening hydrogen bonds and allowing water to enter molecules [35]. Enhancing the CS concentration leads to a more viscous solution that causes an increase in density. A compact polymer structure hinders the uptake of solvent, hence decreasing swelling [36].

### 2.9. Drug Release Study

**Figure 7:**
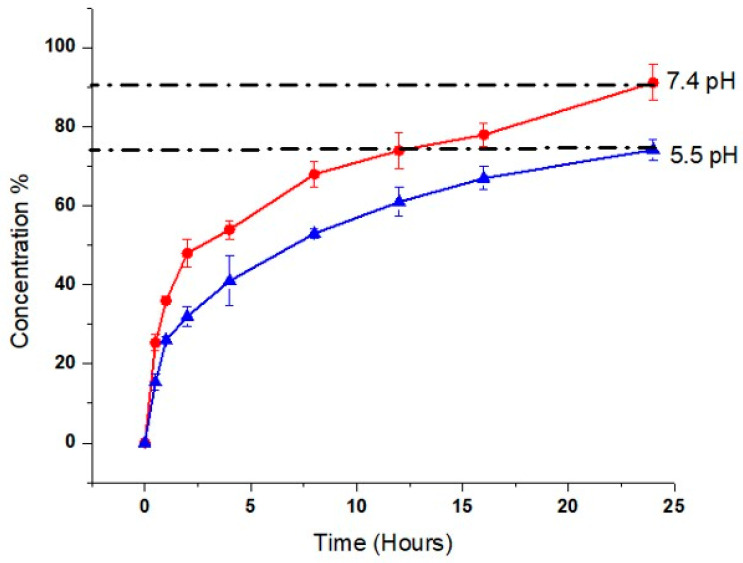
Drug release percentage of the hydrogel film (AM-5) at pH 5.5 and pH 7.4.

By conducting dissolution at pH 5.5 and 7.4 for 24 h at various intervals, the release profile of the model drug (AM) from the hydrogel film was obtained. Sample absorbance measurements were made using HPLC (Shimadzu, Germany) at a specific wavelength (maximum 330 nm). Hydrogel films exhibit higher AM release at pH 7.4 (90.21 ± 1.5)%, (Figure 7), which may be caused by the ionization of the R-COOH (carboxylic) group of MAA. Ionization produces repulsion, which opens spaces for the uptake of water, film expansion, and subsequent AM release [37]. Drug release from the hydrogel film at pH 5.5 is also considerable (74.23 ± 1.7) (Figure 7), according to pharmacopeia. Our findings showed that AM release from the hydrogel film is independent of drug concentration and follows a diffusion process involving the formation of pores in the polymeric matrix (Table 3). As a result, our formulation had the right pore structure for water absorption and drug diffusion from the matrix.

### 2.10. Hemolysis Assay

**Figure 8:**
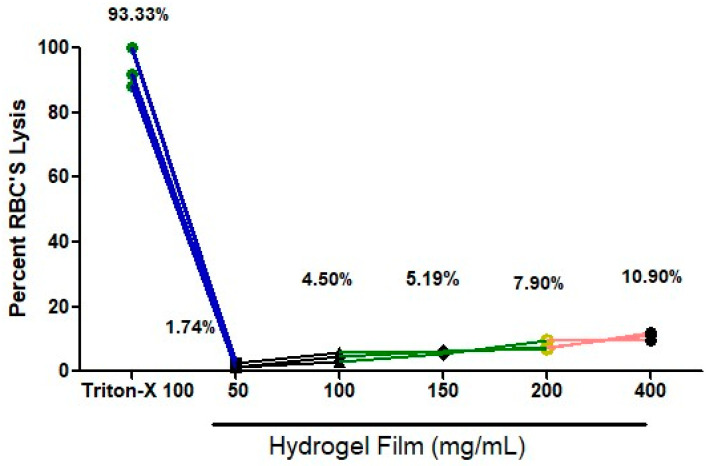
Hemolysis graph showing the biocompatibility of the hydrogel film.

The hydrogel film hemolysis assay was performed as a rapid and accurate way to determine a material’s compatibility with blood [38,39,40,41]. The hemolysis assay measures erythrolysis and hemoglobin dissociation when blood comes into contact with the hydrogel film. After cutting the hydrogel film, weights in mg (50, 100, 150, 200, and 400 mg) were dipped in blood for two hours. After that, a spectrophotometer was used to measure blood compatibility. A graph was created using PBS as the negative control (not shown) and Triton X as the positive control (which had 93.33% lysis, Figure 8). Blood compatibility was measured using different weights, and the results were satisfactory for 50 mg (1.74%), 100 mg (4.50%), 150 mg (5.19%), 200 mg (7.90%), and 400 mg (10.90%). All lysis percentages were below 11% lysis, and for doses of 50 mg, 100 mg, and 150 mg, it was below 6% (Figure 8). According to our research, the hydrogel film could not significantly produce hemolysis. This could be a result of the biocompatible polymer. Due to its flexible mechanical, physical, and biological features and significant capacity to trigger blood coagulation, our results suggest that distinct CS cross-linking with a folic acid hydrogel film has long-lasting hemostatic potential for usage.

### 2.11. Cytotoxicity Study

**Figure 9:**
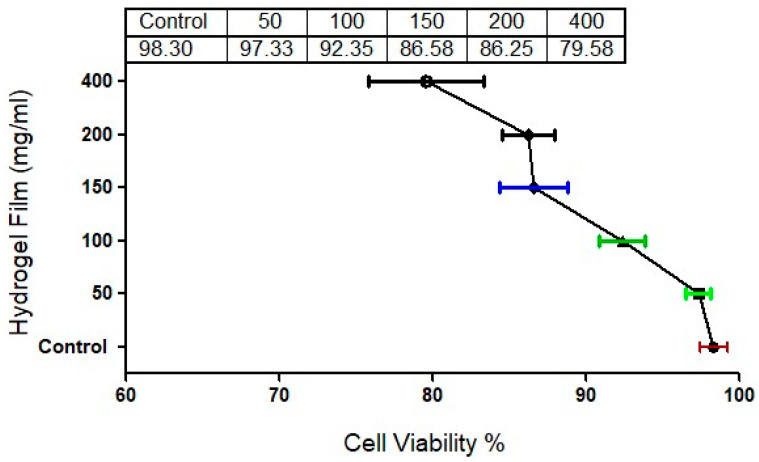
The cytotoxicity of the hydrogel film was measured by MTT assay. The cell viability was determined by calculating the percentage of absorbance in comparison to untreated control cells.

The cytotoxicity of the hydrogel film against the HFF-1 cell lines was examined using the MTT assay (Figure 9). Even after 24 h, the cell viability of the hydrogel film was still more than 98.3 to 79.58%. However, the results demonstrate that the cytotoxicity of the hydrogel film was only slightly raised with high doses, not surpassing 79%, which is necessary for a safe dosage form [42]. This desired cytocompatibility of the hydrogel film may be related to the good cytotoxic nature of chitosan and folic acid reported in the literature [43]. Based on the findings, the developed CS film could serve as a secure platform for wound dressing. 

### 2.12. Microbial Penetration

**Figure 10:**
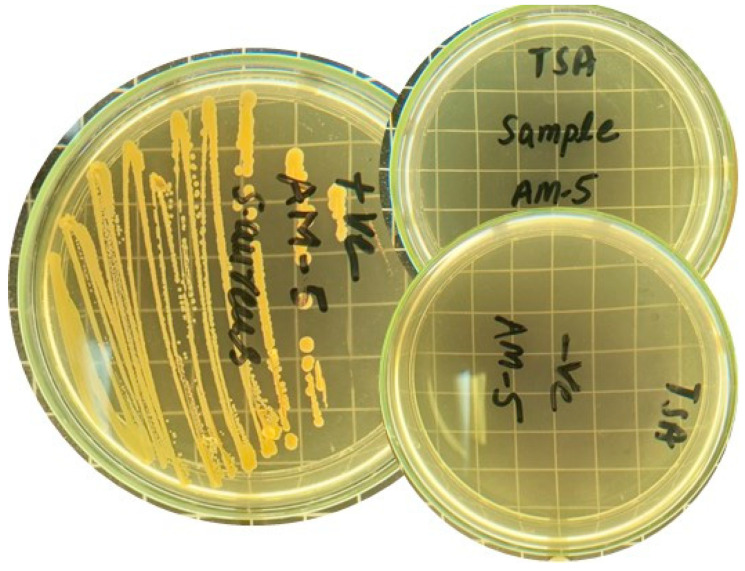
Comparison of AM-5, positive, and negative samples. The positive control exhibited the presence of CFU on the plates.

Wounds produce a moist, warm, and nutritious environment that is perfect for the colonization and proliferation of bacteria. Infections from the outside environment significantly slow the healing of wounds. As a result, dressings for wounds should prevent harmful bacteria from the environment from infiltrating the wound’s surface. That is why film must be microbial-free. To ensure this, an MLT test was used to investigate the microbial load in the hydrogel film. Nutrient agar was used to determine the microbial load of the hydrogel film. A total of 72 h of incubation was performed, and the results showed no microbial growth in the hydrogel-film-containing plates. The positive control, however, displayed CFU in plates, as shown in Figure 10. Table 4 shows the results after some intervals (24, 48, and 72 h).

### 2.13. Antimicrobial Study

**Figure 11:**
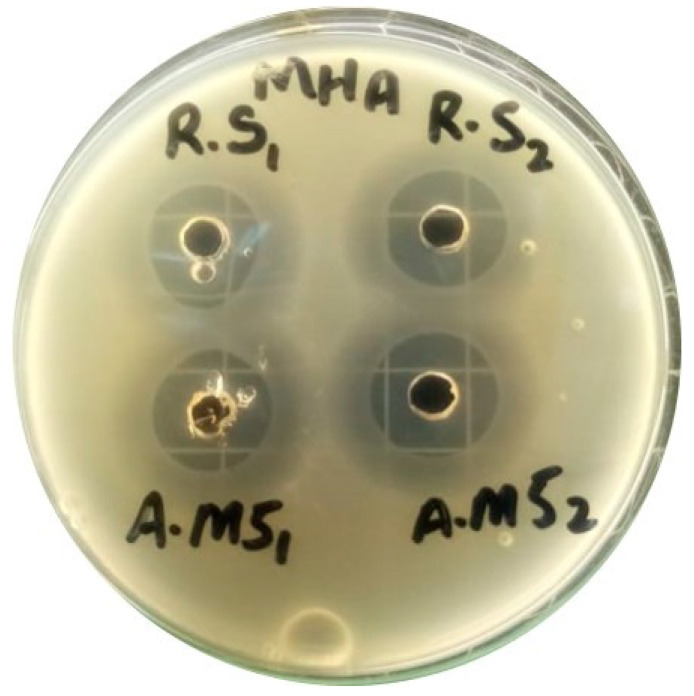
Image of the antibacterial inhibition zones for AM-5 and RS.

The agar well diffusion method was used to assess the in vitro antibacterial effectiveness of hydrogel films against the Staphylococcus aureus bacterium. Figure 11 shows the zone of inhibition for formulation AM-5 along with the reference standard (RS). The AM-based hydrogel film was observed to have strong antibacterial effectiveness and good bacteriostatic properties. The results are consistent with those of other researchers’ reports [44,45]. CS and AM working together synergistically can be used to explain the antibacterial properties of hydrogel film dressings. CS can interact with different kinds of cell membranes because when it dissolves in an acidic environment, the amino groups in the chains protonate into NH^3^+ and become cationic. The primary cause of chitosan’s antibacterial action is its positive charge. It interacts with the negatively charged cell membranes of bacteria, inhibiting their activity or causing cell death [46]. The diameter of the zone of inhibition for the AM-5 hydrogel films against Staphylococcus aureus was 20.62, which is greater than the RS, as shown in Table 5.

### 2.14. Primary Skin Irritation Study of Hydrogel Film

To determine the formulation’s potential for causing irritation, a topical hydrogel film was applied and assessed in accordance with the standards set by the Pharmacy Research Ethics Committee (PREC) of RLCP. We kept an eye on the application site for 24, 36, 48, and 72 h. The rabbit skin exhibited no erythema or irritation after 72 h. The hydrogel film components are therefore safer to apply topically. The topical hydrogel film was shown to have good skin acceptance.

### 2.15. Stability Studies

Stability testing was performed on the hydrogel film formulation (AM-5) in accordance with ICH regulations. The stability study’s test parameters, including thickness, weight variation, folding endurance, moisture content, and moisture uptake, were assessed (Table 6’s data). The produced hydrogel films’ good physical stability during the study period was evidenced by no significant changes in the test parameters that had been chosen.

## 3. Conclusions

By cross-linking chitosan using folic acid, a hydrogel film was successfully developed with amikacin for potential application in wound healing. This study presents a green, easy, and cost-effective procedure for the preparation of a highly effective antimicrobial film that is based on chitosan (CS) hydrogel that has been cross-linked with folic-acid-based molecules. The specialized functional groups of folic-acid-based compounds, known as carboxylate moieties, make them an ideal candidate for electrostatic cross-linking of CS. The hydrogel film showed noticeable swelling, moisture absorption, and mechanical properties, which are important prerequisites for a successful formulation for a wound dressing. The hydrogel film was characterized by FTIR, SEM-AFM, and DSC to investigate the surface morphology and molecular interactions of the polymer utilised to produce the film. Drug release from the hydrogel film and its swelling are directly correlated. Topical film showed no sign of irritation or erythema on the skin after application for up to 72 h. All of the evidence points to CS hydrogel film as a potential delivery system for the drug AM, which is used to treat skin infections. In conclusion, the developed hydrogel films containing amikacin through the cross-linking of chitosan with folic acid have shown promising results for the slow release of the antibiotic AM and potential application as wound dressings for enhancing wound healing.

## 4. Materials and Methods

### 4.1. Chemicals

In this study, Sigma-Aldrich, (Burlington, MA, USA), provided the chitosan (low molecular weight, 50,000–190,000 Da, 75.0% deacetylation) used, as well as the methacrylic acid (MAA) purchased for the experiment. Daejung chemicals & metals Siheung-si, South Korea, provided ammonium peroxodisulfate (APS), methylenebisacrylamide (MBA), and sodium hydrogen sulfate (SHS) for our research. Amikacin (AM) and folic acid were donated for research purposes by Saffron Pharmaceutical in Faisalabad, Pakistan, with 99% purity.

### 4.2. Synthesis

**Figure 12:**
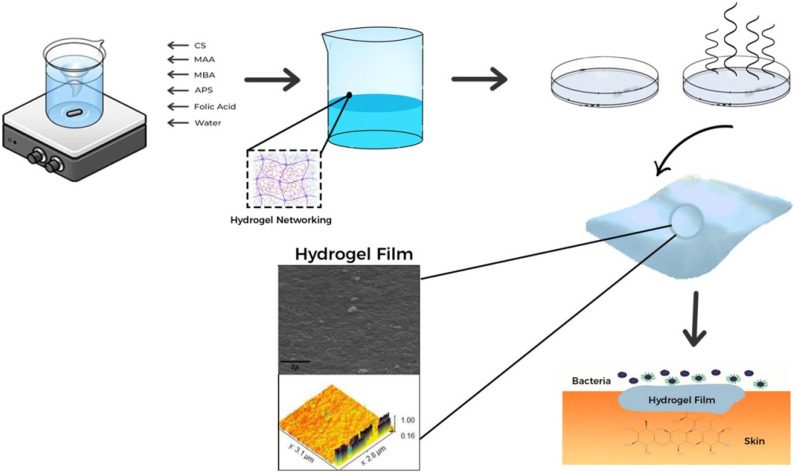
Schematic representation of the synthesis process of the hydrogel film using a free-radical polymerization technique.

In this investigation, a CS-biopolymer-infused hydrogel film was developed (Figure 12) by using a free-radical polymerization technique with varied concentrations of the CS polymer and fixed concentrations of the monomer, initiator, and cross-linker mentioned in Table 7. Folic acid was selected as a green cross-linking agent for chitosan because of its remarkable biocompatibility, high surface functionality, and ability to control drug release. Folic acid also has the added benefit of being environmentally friendly. Both the monomer and the polymer were weighed precisely, as shown in Table 7. Separately, chitosan and MAA were added to clean water and stirred to dissolve them at room temperature 25 ± 2 °C (1500 rpm). At the same temperature of 25 ± 2 °C, the separate monomer and polymer solutions were merged after being dissolved to form a single solution. APS solution was prepared separately in water and added dropwise while being continuously stirred at room temperature 25 ± 2 °C for the polymerization reaction. The cross-linkers folic acid and MBA were dissolved in water to make a solution, which was then slowly added to the reaction solution and named solution I. In step two of the experiment, AM was accurately weighed to 500 mg and dissolved in ethanol (10 mL) along with plasticizers (1 mg) and named solution II. Solution I (40 mL) was measured from the first step of the experiment and mixed with solution II (10 mL). The finished solution was additionally ultrasonically blended to achieve full mixing. To release any trapped oxygen, a stream of nitrogen was given through the reaction mixture. The completed solution was placed into petri dishes, which were then sealed with aluminum foil and cooked for 24 h in a water bath at 50 °C. The petri dishes were removed once the designated time elapsed, and the films were carefully detached from the dried petri plates. Subsequently, the films were stored in desiccators for safekeeping. Five different formulations were created by varying the amount of chitosan. In all experiments, the remaining materials and chemicals stayed the same. We chose the optimum formulation and pursued it for characterization based on the drug-loading property.

### 4.3. Physical Characterization

A digital Vernier caliper (Shang Chong Co., Ltd., Shanghai, China) was used to measure the thickness of the polymeric composite films at various locations with ±0.001 mm accuracy [47]. Five randomly selected samples (n = 5) (2 × 2 cm^2^) were used to determine the weight variation of the films, and the weight of each sample was measured using an electronic weighing balance (OHAUS adventurer Analytical balance, Parsippany, NJ, USA) [48] The number of times a hydrogel film could be folded without cracking or breaking was recorded as the value of each film sample’s folding strength. The folding endurance of the hydrogel films was manually assessed by folding the films repeatedly at the same spot. All parameter results are shown as the mean ± SD [49].

### 4.4. Determination of Drug Entrapment Efficiency

To determine whether drug entrapment was effective, AM-loaded hydrogel film was cut (2 × 2 cm^2^) for 24 h at 37 °C in 10 mL of solvent (ethanol and newly made pH 7.4 phosphate buffer USP, 30:70). AM was extracted using a 20 min sonication procedure. The clear solution was tested with a mobile phase (acetonitrile–sodium acetate buffer (pH 5.0)) to determine the amount of AM using HPLC with a maximum wavelength of 330 nm [24]. Through the following equation, the entrapment efficiency of the formulated hydrogel film for AM was estimated [50].

(1) $$\mathrm{Entrapment}\;\mathrm{efficiency}\%=\;\frac{Actual\;drug\;content\;in\;hydrogel\;film}{Theoretical\;drug\;content\;in\;hydrogel\;film}\;\times \;100$$

### 4.5. Scanning Electron Microscopy and Atomic Force Microscopy

The surface morphology of the hydrogel film was investigated using SEM JEOL analytical electron microscopy. On an aluminum mount, a cut-out hydrogel film sample was sputtered through gold and palladium [51]. Atomic force microscopy has recently become the most often used scanning probe microscope for roughness of hydrogel films. Samples of hydrogel film were analysed by AFM to determine their topography and the roughness of their surfaces. Measurements using an AFM were carried out with the assistance of a Cypher system (AR; Santa Barbara, CA, USA). The experiment was carried out in a laboratory with the temperature set to 25 ± 2 °C and the humidity set to 45 ± 10%.

### 4.6. Fourier Transform Infrared Spectroscopy

This is a technique for identifying the functional groups and determining how closely related pure components are structurally. AM, CS, APS, MBA, folic acid, and loaded dried hydrogel film were used to determine the functional groups and interactions. A Bruker FTIR instrument from the Tensor 27 Series manufactured by Bruker Corporation in Germany was used in conjunction with attenuated-total-reflectance (ATR) technology to obtain the value range of 4000 to 800 cm^−1^ for spectrum scans [52,53,54].

### 4.7. Thermal Analysis

Unloaded and loaded dried hydrogel film and AM were subjected to DSC examination on a thermal analysis device (TA instrument Q2000 Series—West Sussex, UK). In an N_2_ atmosphere, sufficient sample heating was performed at 25 °C per minute up to 350 °C for DSC [55,56].

### 4.8. Powder X-ray Diffraction

PXRD was used to investigate the AM and CS, as well as the composition of the dried hydrogel sheet. An X-ray diffractometer (x-Pert, manufactured by PAN analytical in The Netherlands) was utilised to analyse the samples. The angle of diffraction ranged from 10 to 40° over its entire range [38,39,57,58].

### 4.9. Determination of Gel%, Yield% and Gel Time

To measure the amount of reactant polymerized during the production of the hydrogel film, gel% and yield% were selected. The hydrogel film was properly macerated in water for seven days while being shaken and stirred occasionally to eliminate any polar components. The hydrogel film was first dried in a vacuum oven until it had the same weight throughout. After that, the section of the polymeric network that is impervious to water was baked in an oven until it reached a constant weight (md). By using Equations (2) and (3), gel and yield percent were determined [40].

(2) $$Gel\%=\frac{m_{d}}{m_{i}}\times 100$$

(3) $$Yield\%=\frac{m_{d}}{m_{c}}\times 100$$

### 4.10. Swelling Study

To assess the dynamic swelling and impact of pH sensitivity, the produced hydrogel films were cut, weighed, and then placed in phosphate buffer solution with a pH of 5.5 and 7.4 at a temperature of 37 °C. The medium that generated swelling was regularly removed from the hydrogel film, and the swollen film was weighed at regular intervals until a consistent and uniform weight was attained. The quantification of the normalized swelling degree Q at time t was determined in grams of water per gram of dry gel using the formula below [41].

(4) $$Q_{t}=\frac{m_{t}-m_{o}\;}{m_{o}}$$

*m_t_* = hydrogel film weight after swelling.

*M_o_* = hydrogel film weight before swelling (dry gel).

*Q_t_* = weight of water absorbed.

### 4.11. Release Study

To evaluate drug release at basic pH levels of 5.5 and 7.4, USP dissolution apparatus II was utilised. To keep the drug concentration inside the dissolving medium at a constant level, the hydrogel film was weighed and then added to 900 mL of the dissolution liquid, which was water. The mixture was then constantly spun at 50 revolutions per minute. A temperature of 37 °C was selected as the appropriate temperature for the dissolving medium. Up to 24 h later, samples were collected at predetermined intervals. Every time, fresh medium was used to replace the sampled volume. The maximal AM release was calculated at 330 nm by HPLC [26]. The following formula was used to determine the percentage release.

(5) $$\%\;\mathrm{Release}=\frac{Absorbance\;of\;the\;sample\;solution\;}{Absorbance\;of\;the\;standard\;solution}\;\times \;100$$

### 4.12. Hemolytic Investigations

The supernatant was removed while the precipitate was rinsed three times in phosphate buffer saline (PBS) after being placed in a tube containing ethylene diamine tetraacetic and spun at 1500 rpm for 5 min to perform the hemolytic test on human blood. After thoroughly washing the blood sediment, 200 mL of it was combined with 4 mL of phosphate buffer saline, and the mixture was then vortexed for some time. After the samples had been stored at 37 °C for five hours, the mixture was centrifuged at 1500 revolutions per minute for five minutes. The absorbance of the supernatant at 541 nm was measured. During this experiment, phosphate-buffered saline was employed as the negative control, while Triton X-100 served as the positive control. Microscopically, hemolysis was found in blood cells, and Equation (6) was used to calculate it [59]. All experimental protocols were approved by the Rashid Latif College of Pharmacy (RLCP) Ethical Review Board IRB No (RLCP/EP/107/2023), and the procedures were conducted in compliance with the guidelines outlined in the Declaration of Helsinki by the World Medical Association.

(6) $$\%\;Hemolysis=\frac{ABS\;sample-ABS\;blank}{ABS\;positive-ABS\;blank}\;\times \;100\%$$

### 4.13. Microbial Limit Test

To assess the microbial load in the hydrogel film, microbiological limit test investigations were carried out. For the purpose of determining the microbiological load of the hydrogel film, nutrient agar was utilised. After adding ten millilitres of sterile water to the hydrogel film and properly mixing the ingredients in an area free of microbes, the hydrogel film was ready for use. In glass petri plates, twenty millilitres of agar were poured, and then one millilitre of hydrogel film was placed in the same dish and left to solidify [59]. Then, these plates were incubated for 72 h at 32 ± 2 °C. Colony-forming units (CFU) were calculated on each plate once the period had passed [45]. The measurements and observations underwent three rounds of replication. Petri dishes and nutritional broth were all autoclave sterilized for 30 min at 121 °C prior to the test. The positive control and negative control were also incubated with the sample.

### 4.14. Cell Culture

Human skin fibroblast (HFF-1) (ATCC; SCRC-1041) (species: human; tissue origin: dermal) cells were provided by the University of Lahore in Lahore, Pakistan [59]. These cells were grown in Dulbecco’s modified Eagle medium (DMEM) high-glucose formulation containing 100 mg/mL streptomycin, 100 unit/mL penicillin, and 10% (*v*/*v*) foetal bovine serum. For the experiment, cells were seeded at a density of 1 × 10^4^ per well on 96-well plates and cultivated at 37 °C for 24 to 48 h in an incubator containing 5% carbon dioxide [58].

### 4.15. Cell Viability Assay

The MTT assay was utilised in the process of determining the cytotoxicity of the hydrogel film as well as its biocompatibility. After being rinsed twice with PBS (pH 7.2), the samples were put directly into freshly prepared culture media. After a period of 24 h, a concentration of 10^4^ cells per well was used to cultivate HFF-1 cells that were cultivated in triplicate in 96-well plates that included samples. The control group consisted of cells that did not contain any material. To dissolve the formazan crystals, an additional 150 μL of new culture medium containing 5 mg/mL MTT solution was added to the medium. After that, the MTT formazan precipitate that was purple and blue was dissolved in 200 μL of DMSO. After waiting for 30 min, the absorbance of the solubilized formazan was measured at 570 nm with a multiwell plate reader (Quant Biotek Instruments, Winooski, VT, USA), and the cell viability was estimated with the help of Equation (7).

(7) $$\mathrm{Cell}\;\mathrm{viability}\%=\frac{Mean\;absorbance\;of\;each\;group}{Mean\;absorbance\;of\;the\;control\;group}\;\times \;100$$

### 4.16. Antimicrobial Study

The agar well diffusion method was used to assess the in vitro antibacterial effectiveness of hydrogel films against the Staphylococcus aureus bacterium. Sterile glass petri dishes were used to pour 20 mL of Mueller Hinton Agar/Tryptic Soy Agar from a 100 mL flask into each petri dish; this was allowed to harden into a smooth base layer of uniform depth. After solidification, the petri plates were divided into two equal halves and marked as the sample and standard. Fifty millilitres of medium containing an organism (Staphylococcus aureus) suspension was poured into petri dishes. The hydrogel film sample and reference standard were accurately weighed, dissolved in sterile water, and poured into one hole marked as the reference standard and sample. After filling the holes, the plates were covered with lids and kept smoothly in a hot incubator at 32 ± 2 °C for at least 24 h. After incubation, the results were observed, and the zone of inhibition on plates was measured in mm by Vernier calipers. The results were calculated as per the following formula:

(8) $$\%\;\mathrm{age}\;\mathrm{of}\;\mathrm{AM}=\;\frac{A2\times W1\times P}{A1\times W2}$$

*A*1 = area of zone of inhibition (mm) for standard.

*A*2 = area of zone of inhibition (mm) for sample.

*W*1 = weight of working standard. 

*W*2 = weight of sample. 

*P* = potency of working standard in %.

### 4.17. Skin Irritation Test

The skin irritancy test is important for topical hydrogel films that will be used for dermal application. The capacity of the polymeric cross-linked topical hydrogel film to irritate skin was evaluated using the skin irritation test. The Pharmacy Research Ethics Committee (PREC) evaluated the study’s protocol and gave it their approval (Ethical Review Board IRB No. (RLCP/EP/107/2023)). On the back of a white albino rabbit, where the skin had been made hair-free, the topical hydrogel film was applied in this test. Proper precautions were followed to prevent skin damage when shaving. Within 4 cm^2^, a patch of polymeric cross-linked hydrogel film was placed, and the area was then covered with dressing or tape. Three groups of white albino rabbits, weighing on average 2 ± 0.5 kg, were selected. Group 3 received an unloaded topical hydrogel film (without medication), while Group 1 functioned as the control group and received no therapy. Group 2 received a loaded hydrogel film. The skin was examined 24, 36, 48, and 72 h following application for signs of erythema and oedema. To evaluate the skin reactions, the Draize scale was used [26]. To gauge the severity of the reactions, scores between 0 and 4 were assigned.

### 4.18. Stability Study

According to ICH requirements, the stability of each batch of hydrogel films produced was examined by storing them for three months at a temperature of 40 ± 2 °C and a relative humidity of 75 ± 5%. The hydrogel film samples were deposited in a stability chamber (Remi, India) under the aforementioned circumstances after being covered in aluminum foil. After 3 months, the samples were removed and tested for physicochemical characteristics such as moisture content and folding endurance.

### 4.19. Analytical Statistics

The statistical analysis was carried out with the help of the GraphPad Prism v.5 program. The analysis consisted of one-way ANOVA as well as Tukey’s test. The mean and standard deviation were used to provide a representation of the data (SD). The value of 0.05 for the P statistic was chosen as the cut-off for statistical significance.

## Figures and Tables

**Table 1:** Drug entrapment efficiency of formulations.

Formulation Code	Drug Entrapment Efficiency %
AM-1	54.21 ± 1.51
AM-2	59.12 ± 1.36
AM-3	63.31 ± 1.21
AM-4	71.36 ± 1.41
AM-5	91.81 ± 1.36

**Table 2:** The assessment of the physicochemical properties of hydrogel films (sample size, n = 3).

Formulation	Thickness (mm)	Weight Variation (g)	Folding Endurance	Moisture Content %	Moisture Uptake %
AM-1	0.042 ± 0.02	0.432 ± 0.08	340 ± 11	13.10 ± 1.12	13.10 ± 1.31
AM-2	0.051 ± 0.03	0.471 ± 0.19	359 ± 10	13.70 ± 1.23	14.10 ± 1.42
AM-3	0.059 ± 0.05	0.522 ± 0.07	410 ± 19	15.20 ± 1.34	15.10 ± 1.42
AM-4	0.062 ± 0.02	0.512 ± 0.04	430 ± 13	16.10 ± 1.16	16.10 ± 1.32
AM-5	0.074 ± 0.04	0.416 ± 0.29	490 ± 11	16.80 ± 1.27	16.70 ± 1.23

**Table 3:** Drug entrapment efficiency (all formulations) and percent drug release at pH 5.5 and pH 7.4 (AM-5).

Code	Drug Entrapment Efficiency	Percent Release (for a 24 h Period)	
		pH 5.5	pH 7.4
AM-1	54.21		
AM-2	59.12		
AM-3	63.31		
AM-4	71.36		
AM-5	91.81	74.23 ± 1.7	90.21 ± 1.2

**Table 4:** Results of microbial penetration test for hydrogel films.

Time (h)	Positive (Control)	Hydrogel Film	Negative
24	No	No	No
48	Yes	No	No
72	Yes	No	No

**Table 5:** Zone of inhibition of RS and AM-5 hydrogel film.

Zone of Inhibition (mm)		
Assay #	Reference solution	AM-5 solution
1	20.42	20.53
2	20.62	20.72
Average	20.52	20.62
% Assay		Limit% 90–110
Calculation		$\frac{A_{2}\times W\times 10\times 50\times 50\times P\times 2\times 100}{A_{1}\times 50\times 25\times V\times 10\times 1000\times 100}$
Reference solution (A_1_)		20.62
AM-5 solution (A_2_)		20.52
Weight of reference stand (W)		72.20
AM-5 film mixed in water (V)		2.00 mL
Potency of reference (P)		692.50 μg/mg
% assay of amikacin		99.51% ± 2.00%

**Table 6:** Physicochemical parameters of the optimised hydrogel films after the stability period.

Formulation	Thickness (mm)	Weight Variation (g)	Folding Endurance	Moisture Content %	Moisture Uptake %
AM-5	0.078 ± 0.14	0.496 ± 0.21	499 ± 12	16.93 ± 1.87	16.97 ± 1.13

**Table 7:** Composition of formulations, step I and step II.

Formulation Code	Chitosan wt%	Folic Acid wt%	MAA wt%	APS wt%	MBA wt%	AM mg
AM-1	0.4	0.4	20	0.32	0.1	500
AM-2	0.8	0.4	20	0.32	0.1	500
AM-3	1.2	0.4	20	0.32	0.1	500
AM-4	1.6	0.4	20	0.32	0.1	500
AM-5	2.0	0.4	20	0.32	0.1	500

## Data Availability

All the raw data of this research can be obtained from the corresponding authors upon reasonable request.
